# Transactional relationships and sex with a woman in prostitution: prevalence and patterns in a representative sample of South African men

**DOI:** 10.1186/1471-2458-12-325

**Published:** 2012-05-02

**Authors:** Rachel Jewkes, Robert Morrell, Yandisa Sikweyiya, Kristin Dunkle, Loveday Penn-Kekana

**Affiliations:** 1 Gender & Health Research Unit, Medical Research Council, Pretoria and School of Public Health, Faculty of Health Sciences, University of the Witwatersrand, Johannesburg, South Africa; 2 Research Office, University of Cape Town, Cape Town, South Africa; 3 Gender & Health Research Unit, Medical Research Council, Pretoria, South Africa; 4 Rollins School of Public Health, Emory University, Atlanta, USA; 5 Centre for Health Policy, Faculty of Health Sciences, University of the Witwatersrand, Johannesburg, South Africa; 6 Gender & Health Research Unit, Medical Research Council, Private Bag X385, Pretoria, 0001, South Africa

**Keywords:** Transactional sex, Prostitution, Commodified sex, South African men, Epidemiology, Sexual entitlement

## Abstract

**Background:**

Sex motivated by economic exchange is a public health concern as a driver of the Sub-Saharan African HIV epidemic. We describe patterns of engagement in transactional sexual relationships and sex with women in prostitution of South African men, and suggest interpretations that advance our understanding of the phenomenon.

**Methods:**

Cross-sectional study with a randomly-selected sample of 1645 sexually active men aged 18–49 years who completed interviews in a household study and were asked whether they had had sex with a woman in prostitution, or had had a relationship or sex they took to be motivated by the expectation of material gain (transactional sex).

**Results:**

18% of men had ever had sex with a woman in prostitution, 66% at least one type of transactional sexual relationship, only 30% of men had done neither. Most men had had a transactional relationship/sex with a main partner (58% of all men), 42% with a concurrent partner (or *makhwapheni*) and 44% with a once off partner, and there was almost no difference in reports of what was provided to women of different partner types. The majority of men distinguished the two types of sexual relationships and even among men who had once-off transactional sex and gave cash (n = 314), few (34%) reported that they had had sex with a ‘prostitute’. Transactional sex was more common among men aged 25–34 years, less educated men and low income earners rather than those with none or higher income. Having had sex with a woman in prostitution varied little between social and demographic categories, but was less common among the unwaged or very low earners.

**Conclusions:**

The notion of ‘transactional sex’ developed through research with women does not translate easily to men. Many perceive expectations that they fulfil a provider role, with quid pro quo entitlement to sex. Men distinguished these circumstances of sex from having sex with a woman in prostitution. Whilst there may be similarities, when viewed relationally, these are quite distinct practices. Conflating them is sociologically inappropriate. Efforts to work with men to reduce transactional sex should focus on addressing sexual entitlement and promoting gender inequity.

## Background

Sex motivated by economic exchange is associated with public health problems, and presents many conceptual challenges for researchers. Women and men in prostitution are vulnerable to HIV and other sexually transmitted infections, as well as acts of violence from clients, pimps, police and other parties [1-5]. A similar set of vulnerabilities pertain amongst women who engage in transactional sex [6,7]. Whilst the categories of ‘prostitution’, ‘sex work’ and ‘transactional sex’ are at times deployed in public health literature as if their meanings were evident, these are very complex and highly contested [8].

The complexity of what is often referred to as ‘sex work’ is highlighted in a paper by Harcourt and Donovan, who describe 25 ‘types of sex work’ [9]. Transactional sex is open to multiple definitions, and many authors fail to define it [8,10]. This is essential in quantitative research, and the definition used in such work has generally been sex that is predicated on actual or anticipated material gain (instrumental support such as transportation or a place to sleep, material goods or cash) [6]. Whilst the definition may be rendered with some ease into items that can be asked of those receiving the goods (usually women), it is much harder to assess when questioning those who ‘give’ because the notion of ‘predicated’ is based on perception and perceptions of motivation may differ between parties in a sexual encounter, and indeed may be fluid. A further area of definitional contestation is the intersection between transactional acts and transactional relationships. The former may occur within relationships that are predominantly based on other factors, which may include love, marriage, emotional support, habit or social expedience. Material gain is sometimes the only factor sustaining a relationship, but it is more common for multiple factors to be involved (even negative factors such as fear) and it can be difficult to distinguish between male fulfilment of a traditional provider role and relationships that could not be sustained if that provider role was not fulfilled. A notion of ‘transactional sex’ fits poorly in a context where customary or legal conventions are in place, for example, in the context of marriage. In contexts where the borders between transactional sex and customary wooing practices (which are considered part of a process that leads to marriage and involve gift-giving [11,12]) are shifting, definitional clarity is difficult to find. In South Africa, the conceptualisation of ‘transactional relationships’, whether with main partners (ie women who are not legally considered as wives but who in effect enjoy some of the status and benefits) or *makhwapheni* [plural, a term in Nguni languages for an indigenous category of secondary concurrent partners], may shift.

Transactional relationships, sex and prostitution may be thought of as lying on a spectrum. At one end, transactional sex may have very many apparent similarities with the practice of prostitution, particularly when cash is given by a sexual partner after a single act of sex, or where there is a ‘relationship’ involving multiple sexual encounters that is entirely sustained by receipt of material reward. Boundaries may be extremely hazy, when acts and context may differ little. Transactional sex may include negotiated cash for sex exchange [10,13], although in South Africa this is not often reported. Except where women are actively soliciting sex in a public area, in a brothel or embrace an identity as a ‘prostitute’ or ‘sex worker’, it may be very hard to distinguish between transactional sex and prostitution at one end of the spectrum. However, usually there is no explicit negotiated exchange with transactional sex, and a wide range of goods or services may be received. Indeed at the other end of the spectrum, it can be very hard to distinguish transactional relationships from other relationships where there is a predominant expectation by both the man and the woman that one partner (usually the man) will fulfil a provider role, especially as the role of romantic love in sustaining relationships is complex, fluid and may be very closely linked to expressions as gifts [11]. Interpreting the spectrum is rendered more difficult by literature that shows that in many parts of sub-Saharan Africa it is sex given *without* material reward that is perceived to be demeaning for women [14,15].

Transactional sex and relationships are often cited to demonstrate that the commodification and instrumental use of sex is culturally normative in modern day South Africa [11,14], in a way that may be different from some European and higher income countries. Some authors have cogently argued that central elements of transactional sex are ‘a moral obligation to support the needy’ [16] and have drawn connections with widow inheritance and historical pressure on wealthy men to have several wives [14]. This is particularly pertinent at a time of review of legislation on adult prostitution. In high income countries, there is traditionally very little non-stigmatized social space for acknowledging transactional relationships or sexual encounters in which sex is exchanged for material reward [17]. While recent research from the United States suggests that economically motivated relationships and transactional sex are in fact common and reported by one in three unmarried US women [18], the social stigma surrounding disclosure of such relationships is much more pronounced than in Southern Africa [17]. In South Africa, there is a divergence in views on commodification of sex. Some perceive *lobola* (bride price) and cultural practices of widow inheritance as normalising the commodification of sex [11], but others challenge this interpretation of African tradition (e.g. Guy [19]). It is also at variance with a common discourse rooted in Christian-influenced morality which emphasises procreative sex and places sex in marriage and other committed relationships [11]. Because South Africa is a complex, multi-racial, multi-ethnic and multi-cultural country, the arena of material exchange and sex across all groups needs to be better understood to better inform both public health and public policy interventions.

Despite concern about transactional sex and prostitution and growing recognition that they are both important in the HIV epidemic, it is an area that has been generally under-researched and under-theorised. Knowledge about men who have sex with women in prostitution and those who provide resources for women in the course of transactional relationships (or sex) is often missing from research in this area and from public health and policy debates. Yet men are very important. Some authors on prostitution argue that the entire sex industry is shaped by sex-buying men [20], notwithstanding the importance of the forces (such as poverty and drug use) that drive women towards engagement in prostitution or the role of supply in stimulating demand [21]. The limited focus on men in transactional sex research restricts the extent to which the phenomenon can be understood relationally which has the danger of inclining analysis to a one-sided examination of the phenomenon. Whilst one study with male youth participating in an HIV prevention program in the Eastern Cape province of South Africa found that 18% reported transactional sex with casual partners and 15% with main partners [7], the overall extent and patterns of engagement in transactional relationships/sex and women in prostitution among adult men in the general population in South Africa is not known. Given that South Africa is currently engaged in a review of its laws on prostitution, and that legislators perceive that legal deliberations should be informed by research, it’s very important to generate research evidence to better understand this area.

To explore these issues, we draw on data from a large population-based household survey conducted with a randomly selected sample of adult South African men which included questions on transactional sexual relationships and sex with women in prostitution. We examine the prevalence and patterns of men’s self-reported engagement in transactional sexual relationships with women and sex with women in prostitution across social and demographic characteristics.

## Methods

Data were collected from three districts in the Eastern Cape and KwaZulu-Natal provinces of South Africa. These form a contiguous area, and include rural areas with communally-owned land under traditional leadership, as well as commercial farms, villages, towns, and a city, inhabited by people of all South African racial groups, several ethnic groups (predominantly Xhosa and Zulu) and socio-economic backgrounds.

The sample used a two stage proportionate stratified design to identify a representative sample of men aged 18–49 years living in the three districts. Using the 2001 census as the primary sampling frame, 222 census enumeration areas (EAs) were selected as the primary sampling unit, stratified by district and with numbers proportionate to district population size. The sample was drawn by Statistics South Africa. Households in each EA were mapped and twenty were systematically selected. In each household one eligible man was randomly selected to take part in the interview. Men were eligible for the study if they were aged 18–49 years and had slept there the night before.

Of the 222 selected EAs, two (0.9%) had no homes, and in five (2.3%) we could not interview because permission from the local political gatekeepers was declined (1) or we could not access any eligible home after multiple visits at different times of day (4). In all the latter EAs, we established that many households were ineligible due to age or absence of a man. We completed interviews in 215 of 220 eligible EAs (97.7%). We sampled a total of 4473 visiting points. Of these, 1353 (37.1%) were found to contain no eligible man, 2298 (51.4%) contained at least 1 eligible man, and 822 (18.4%) could not be rostered for eligibility after a minimum of 3 attempts at contact. We completed interviews in 1737 of 2298 (75.6%) enumerated and eligible households. This analysis is based on 1645 sexually active men who provided information on transactional sex and having had sex with women in prostitution.

Interviews were conducted in isiXhosa or isiZulu or English with data collected using self-completion on APDAs (Audio-enhanced Personal Digital Assistants). Each participant could hear and read each question and its response options in the language of his choice, and enter his answer directly into the APDA. Interviews thus allowed for totally anonymous reporting and took 45–60 minutes to complete.

### Measurement

The questionnaire included categorical variables measuring age, education, race, employment, marital status and income. To measure transactional sex with women, men were asked separately for main partners and on-going secondary partners (*makhwapheni* (pl.) in isiZulu and isiXhosa) “Do you think any of them became involved with you because they expected you to do, or because you did do any of the following:” with yes/no response options for providing food, clothes, cell phone or transportation; school fees or residence fees; somewhere to stay; cosmetics; items for children or family; handyman work; cash or money to pay bills; and anything else that she could not afford by herself. For once-off partners, men were asked “Have you ever had sex with a woman just as a once off because you gave her or she expected that you would give her:” with response options for food, clothes, or cosmetics; transportation; a place to sleep for the night; handyman work; cash or money to cover expenses; and anything else that she could not afford by herself. A man who responded affirmatively to any item for each partner type was considered to have engaged in a transactional relationship or (in the case of a once-off) transactional sex with that type of partner. A separate yes/no item asked men “Have you ever had sex with a prostitute?” For the analysis presented here, men who have never had sex or never had a partner of a particular type were coded as not having done that particular type of sex, based on an assumption that that man could have, had they offered material reward for it.

### Ethical issues

The men were given an information sheet about the study and signed informed consent separately for the interview and for providing a dried blood spot sample for HIV antibody testing (not discussed here further as data not presented). As an incentive, they were given R25 (US $3.2) for the interview and those who gave a blood sample for HIV testing were given a further R25 (data not discussed in this paper). Since the questionnaire asked men to disclose a range of criminal acts and South African law does not privilege research data, interviews were conducted anonymously using the APDAs and no identifying details of the men or their households were recorded or retained. This precaution meant that the consent forms could not be linked to individual data points. Ethics approval was given by the Medical Research Council’s Ethics Committee.

### Data analysis

The study design provided a self-weighted sample. Data files were collated from APDAs. Analyses were carried out using Stata 10.0. All procedures took into account the two stage structure of the dataset, with stratification by district and the EAs treated as clusters. The distribution of patterns of transactional relationships or sex, items provided, and the prevalence of transactional relationships or sex and having had sex with a woman in prostitution were summarised by social and demographic characteristics as percentages, with 95% confidence limits calculated using standard methods for estimating confidence intervals from complex multistage sample surveys (Taylor linearization). Pearson’s chi was used to test associations between categorical variables. No efforts were made to replace missing data.

We next constructed two logistic regression models, one showing factors associated with having ever had a transactional relationship or sex and the other factors associated with having had sex with a woman in prostitution. To account for clustering of men within EAs, we treated EAs as random effects within the models. Significant social and demographic variables were tested in the models, as well as a term for stratum, without elimination of non-significant variables.

## Results

The social and demographic characteristics of the sample are presented in Table 1. Half of the men were under the age of 25 years old, and over 80% were under 35. The majority (60%) had not completed school and nearly one in five men had no secondary school education. The men were drawn from all South Africa’s main racial categories, but most were Black African (85%), with 9% Indian, 4% Coloured and 2% White. Nearly half of the men were unemployed and had no monthly income. Most of the others had very low income, with 39% earning between R1-R2000 per month. Only 5% of the men had an income over R5000 per month. Most of the men (62%) were single, with fewer than one in four married (23%). A further 11% were cohabiting.

**Table 1:** Social and demographic characteristics of the sample and prevalence of having had sex with a woman in prostitution or a transactional relationship or sex

	**n**	**N**	**%**	
**Age:** 18-24	858	1645	52.1	
25-34	508	1645	30.9	
35-49	279	1645	17.0	
**Education:** no more than primary	310	1636	18.9	
Secondary	670	1636	41.0	
Matric or higher	656	1636	40.1	
**Race:** African	1401	1645	85.2	
Coloured	70	1645	4.3	
Indian	148	1645	9.0	
White	26	1645	1.6	
**Monthly income:** none	742	1524	48.7	
R 1-500	266	1524	17.5	
R 500 - 2000	320	1524	21.0	
R 2001- 5000	115	1524	7.5	
R 5000 +	81	1524	5.3	
**Marital status:** married	358	1553	23.1	
cohabiting	176	1553	11.3	
divorced/widowed	55	1553	3.5	
single	964	1553	62.1	
**Prevalence of engagement with transactional relationships and sex with a woman in prostitution**				**95% CI**
Ever had sex with a woman in prostitution	296	1615	18.3	16.2, 20.7
Transactional relationship or sex with any partner	1084	1645	65.9	63.1, 68.6
Transactional relationship with a main partner	953	1643	58.0	55.2, 60.7
Transactional relationship with a khwapheni	678	1626	41.7	38.8, 44.7
Transactional sex with a once off partner	711	1621	43.9	40.7, 47.0

**Figure 1:**
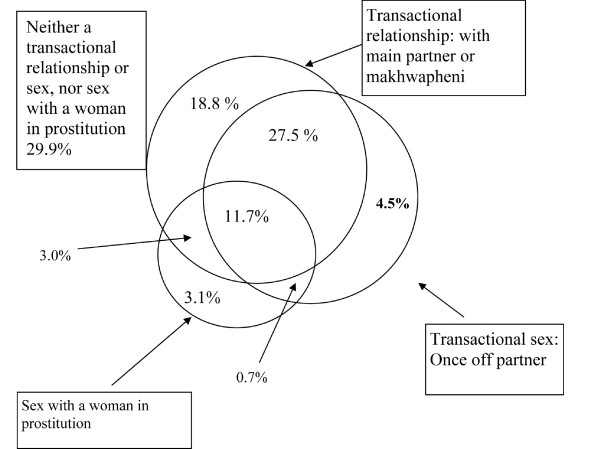
Venn diagram showing the overlap between self-reported engagement in different forms of transactional relationship or sex and having sex with a woman in prostitution.

The prevalence of having self-reported a transactional relationship or sex with different types of partners or sex with a woman in prostitution is shown in Table 1. Overall, 18% of men reported ever having sex with a woman in prostitution, while two thirds (66%) reported at least one type of transactional sexual relationship. The commonest partner with whom a transactional relationship/sex was reported was a main partner (58% of the whole sample), with similar proportions of men disclosing a transactional relationship with a *khwapheni* (42%) or once off partner (44%). Figure 1 shows the overlap between the different types of men’s transactions. A minority of men disclosed no transactional sex or relationship, nor sex with a woman in prostitution (29.9% of all men). The most commonly reported pattern was of having had one or more transactional relationships (with a main partner or *khwapheni*) and transactional sex with a woman as a once off (39.2% of total) and/or sex with a woman in prostitution (14.7% of total). Among the men reporting transactional relationships with a main partner or *khwapheni*, less than a third (30.8%) had had these without also having sex with a woman in prostitution or transactional sex with a once off partner. The group of men who had had sex with a woman in prostitution without also having a transactional relationship or once-off transactional sex was very small (3% of the total sample) but constituted a quarter of men who had had sex with a woman in prostitution. Whilst there was considerable overlap between reports of having had once-off transactional sex and sex with a woman in prostitution, 4.5% of the men reported the former but not the latter. Among men who had once-off transactional sex and gave cash (n = 314), only 34.4% reported that they had had sex with a ‘prostitute’.

Table 2 shows the range of goods and services exchanged for sex with different partner types. The most striking feature of the table is that there was very little difference in what men gave women by type of partner. The one exception is the reciprocation of once-off sex when a bed or lodging was offered. Most men reported transactions across a range of different categories, suggesting that either they had transactions with many partners, or more likely that more than one item was often given or anticipated. The most common transactions involved money. Half of all men reporting transactional relationship/sex reported giving cash to a partner irrespective of partner type, but the proportion was probably much higher as generally gifts of cosmetics, airtime etc take the form of cash for these items. For main partners and *khwapheni*, the most commonly reported items were money for cosmetics and beauty products, whereas for once-off partners it was most commonly a place to sleep.

**Table 2:** What did they give? The prevalence of goods given to partners by men reporting each type of transactional sexual relationship

	**Transaction with female main partner**	**Transaction with khwapheni**	**Transaction with once-off partner**
	**(N = 953)**	**(N = 678)**	**(N = 711)**
Provided her with food, clothes, cell phone or transportation	66.9% (635)	58.8% (395)	
Paid her school fees or residence fees	36.7% (349)	36.7% (245)	
Provided her with somewhere to stay	43.1% (408)	42.1% (281)	72.6% (513)
Gave her cosmetics or money for beauty products	72.3% (683)	71.5% (476)	
Gave items for her children or family	41.9% (395)	38.2% (255)	
Gave her cash or money to pay her bills	49.2% (466)	48.5% (320)	45.0% (317)
Provided her anything else that she could not afford by herself	58.3% (553)	56.8% (374)	53.1% (372)
Did handyman work for her or fixed her car	26.3% (247)	28.4% (187)	29.7% (207)
Food, clothes or cosmetics	Not asked	Not asked	55.9% (393)
Lift, ticket or money for transport	Not asked	Not asked	63.2% (448)

The prevalence of transactional relationships or sex was very high in all social and demographic categories. However, there were significant differences between the age groups, with highest prevalence among men aged 25–34 years compared to older or younger men (Table 3). This age group was also significantly more likely to report sex with a woman in prostitution. There were also significant differences in the prevalence of both behaviours by race. Coloured men were most likely to report both transactional sex and sex with a woman in prostitution. Among men from other race groups, Black African men were most likely to report transactional sex but least likely to report sex with a woman in prostitution. Indian men were less likely than Black African men to have transactional sex, but were more likely to report sex with a woman in prostitution. Education had no association with reporting transactional sex, but increasing education was associated with increasing likelihood of reporting sex with a woman in prostitution. For both transactional sex and sex with a woman in prostitution, men with no income were least likely to report, although over 61% still reported transactional sex, only 13% had had sex with a woman in prostitution. Transactional sex or relationships were most prevalent among very low income men (earning R1- 2000 per month) whereas sex with a woman in prostitution was found among the slightly higher earners (over R500 per month) and in the higher income categories. Cohabiting, divorced or widowed men were more likely to report both behaviours.

**Table 3:** The prevalence of having had a transactional relationship or sex, or sex with a woman in prostitution among men by socio-demographic characteristics

	**Any transactional relationship/sex (%)**	**95% CIs**	**p value**	**Sex with woman in prostitution (%)**	**95% CIs**	**p value**
**Age:** 18-24	60.4	56.9, 63.9	<0.0001	16.1	13.4, 18.7	0.012
25-34	75.2	71.1, 79.3		22.4	18.4, 26.4	
35-49	65.9	60.3, 71.6		17.8	13.2, 22.4	
**Education:** no more than primary	71.3	66.0, 76.6	0.077	13.1	9.2, 16.9	0.014
secondary	63.9	59.7, 68.1		18.3	15.3, 21.4	
Matric or higher	65.4	61.5, 69.3		21.0	17.4, 24.7	
**Race:** African	67.0	64.0, 69.9	0.0036	16.3	14.1, 18.6	0.0001
Coloured	75.7	64.6, 86.8		38.2	24.2, 52.3	
Indian	54.1	43.9,64.2		26.4	19.4, 33.3	
White	50.0	30.8, 69.2		26.9	3.1, 50.7	
**Monthly income:** none	61.1	57.2, 64.9	<0.0001	13.0	10.4, 15.6	<0.0001
R 1-500	71.4	65.7, 77.2		15.3	10.9, 19.7	
R 500 - 2000	76.3	71.5, 81.0		27.9	22.5, 33.3	
R 2001- 5000	65.2	56.3, 74.1		31.3	21.5, 41.0	
R 5000 +	61.7	50.7, 72.8		29.2	18.8, 39.7	
**Marital status:** married	60.9	55.6, 66.2	0.0001	16	12.1, 19.9	0.002
cohabiting	79	73.1, 84.9		30.2	0.22.0, 38.5	
divorced/widowed	78.2	67.0, 89.4		20	8.2, 31.8	
single	64.9	61.4, 68.5		17.5	14.9, 20.2	

Logistic regression models of social and demographic factors associated with having had a transactional relationship or sex, and sex with a woman in prostitution are given in Table 4, and show both interesting similarities and differences when compared with two-way associations. Older men (over 24 years) were significantly more likely to report transactional sex than younger men, although this was marginal for men aged 35‐49 years (p = 0.06). Men with only primary school education were more likely than those with secondary education, Black African men were more likely than Indian men, those earning under R2000 per month were more likely than those with no income, and cohabiting, widowed or divorced men were more likely than married men to have had transactional relationships or sex.

**Table 4:** Logistic regression models of social and demographic characteristics associated with having had a transactional relationship or sex, or sex with a woman in prostitution

	**Any transactional relationship or sex**			**Sex with woman in prostitution**		
	**OR**	**95% CI**	**P value**	**OR**	**95% CI**	**P value**
**Age:** 18-24	1.00			1.00		
25-34	1.90	1.42, 2,56	<0.0001	1.17	0.84, 1.63	0.361
35-49	1.43	0.98, 2.08	0.064	1.03	0.65, 1.63	0.907
**Education:** no more than primary	1.00			1.00		
Secondary	0.67	0.47, 0.96	0.029	1.25	0.79, 1.97	0.345
Matric or higher	0.71	0.49, 1.04	0.082	1.25	0.78, 2.00	0.363
**Race:** African	1.00			1.00		
Coloured	1.15	0.59, 2.22	0.685	2.14	1.16, 3.95	0.015
Indian	0.53	0.33, 0.84	0.008	1.20	0.74, 1.96	0.462
White	0.47	0.19, 1.17	0.106	1.49	0.56, 3.95	0.422
**Monthly income:** none	1.00			1.00		
R 1-500	1.64	1.17, 2.30	0.004	1.24	0.81, 1.91	0.327
R 500 - 2000	1.89	1.35, 2.65	<0.0001	2.09	1.46, 3.01	<0.0001
R 2001- 5000	1.11	0.68, 1.82	0.672	2.20	1.30, 3.73	0.003
R 5000 +	1.22	0.68, 2.19	0.516	2.03	1.06, 3.87	0.032
**Marital status:** married	1.00			1.00		
cohabiting	1.86	1.15, 3.00	0.011	2.08	1.27, 3.41	0.004
divorced/widowed	2.23	1.06, 4.72	0.035	1.75	0.80, 3.81	0.161
single	1.25	0.92, 1.70	0.155	1.22	0.82, 1.81	0.324

The logistic regression model suggests that the odds of having had sex with a woman in prostitution vary little between social and demographic categories. It was more common among Coloured than Black African men, among cohabiting than married men and among men earning over R500 per month.

## Discussion

The prevalence of men having what we have referred to as a transactional sexual relationship, i.e. where they perceive that the relationship or sex act was predicated on a provider expectation (whether fulfilled or not), was very high at 66%, and much higher than the prevalence of having had sex with someone they identified as ‘a prostitute’ (18%). Through asking about transactional relationships or sex with different types of partners (although not with wives), we have shown that almost all men who ever report these, have such relationships with their main partners, and further that there is almost no difference in the nature of the provider expectation between partner types. We acknowledge however that our question formulation required men to speculate about their sexual partner’s motives, but in so doing we measured their perceptions of expectations. This very strongly suggests that a large group of men within the South African population perceive themselves to be expected to provide for their partner in various ways within a relationship and that they would not have an opportunity for that relationship if they did not fulfil that role [11]. Most explicit in the case of transactional sex with once off partners, but also otherwise implicit, given that what men reported were sexual relationships with other women, is the idea that by so doing men are entitled to sex with this partner i.e. on the woman’s part receiving entails obligation.

The study findings suggest that in as far as we have attempted to measure and study transactional relationships and sex, what we have succeeded in capturing may best be understood as a conservative notion of male gender roles in relationships, where men are expected to provide and women in return are sexually available. This has been well described in qualitative work from Africa (e.g [22-24]). Clearly this notion prevails in all of the social groups studied, albeit with some difference in frequency, but is most dominantly seen among low income, lower educated, Black African and Coloured men. This sits uncomfortably with the notion of a category of ‘transactional sex’ or even ‘transactional relationships’ although we have used such terminology in this paper. These notions suggest a certain instrumentality that individual men may apply to some relationships or sexual acts but not others, whereas it seems we have rather tapped into a provider notion which would probably apply across all these relationships. The notion of transactional sex has predominantly been developed and utilised through research with women, some of which captures a very particular instrumentality aspired to in sexual encounters or relationships [16,25,26]. An extreme example is the somewhat mythical ‘3C’s girl, exploiting men for cash, cars and cellphones [8]. We would like to suggest that women’s discourse of transactional relationships and sex may appear empowered and liberal, and thus contrast with men’s perceptions of provider expectations, which are a reference to a very conservative gender norm, but there may often be a clash in reality. If conservative gender norms generate expectations that men will provide for women, then it is possible for a women to feel empowered by ‘exploiting men’ (to use the language of one of Leclerc-Madldla’s informants [8]), whilst the ‘exploited men’ view themselves in a conservative gender role. Thus this agentic expression of a ‘modern’ feminine position comes at a particular cost in terms of complicity with the patriarchal gender order (c.f Jewkes, Morrell and Haram [12,23]).

The study findings suggest that many men perceive that they are expected to provide a considerable range of goods to their women partners. Whilst this list included some items that could be rendered in kind (such as repairs), by and large the men indicated that the realm of sexual relationships is cash economy. It is very easy to see how these social expectations put pressure on men, especially in context of unemployment, and may be strongly resented by men who have little or no money. Further this gives rise to circumstances in which men may mistrust the motivations and commitment of women sexual partners (c.f. Mganja et al [22]).

Whilst it was earlier suggested that transactional sex blends with sex with a woman in prostitution, it seemed that many of the men did not see it that way. The manner in which men responded to the questions indicated that they distinguished between sexual relationships and acts that revolved around providing and reciprocal sex, from sex with a woman in prostitution. This was most clearly seen in the reports of transactional sex with a once off partner where the man provided cash. To an outsider, this may appear remarkably similar to the type of transaction found in prostitution, but most men made a distinction. In fact two thirds of men who described having been in such a situation said they had not had sex with a prostitute. This may be because having sex with a woman in prostitution is largely stigmatised whereas transactional sex is regarded as ‘normal’ and acceptable (by both men and women) [10,17,27].

There were racial differences, with a very much lower proportion of Black African men reporting having had sex with a woman in prostitution than Coloured and Indian men, although an important part of this difference was due to many Black African men being of lower income. Although there were significant differences in both the prevalence of transactional sex and sex with a woman in prostitution between income categories, the differences were largest when it came to sex with a woman in prostitution. This has been a relatively infrequent experience for men who were not earning, compared to those earning R500 of more per month. The prevalence of transactional sex was very high for all income categories and the proportional difference between categories was not large, suggesting that expectations of men fulfilling provider roles are relatively inelastic when related to income. The findings that very low income men have paid for sex is in keeping with observations of the sex industry internationally [28] and findings that low income men may spend a greater proportion of their income on casual partnerships than higher income men [29].

The prevalence of sex with a woman in prostitution in this study was nevertheless higher than that reported from Sweden (18% v. 13%) [30,31] cited in [32]. But in both countries it cuts across all ages and income classes. In South Africa, like elsewhere, many men who have had sex with a woman in prostitution are married or cohabiting, although the cross-sectional nature of the research prevents us from knowing their relationships status at the time when they did it.

We have shown that men who are older (from their mid-twenties) are more likely to have had transactional sex, but there was no association between age and having sex with a woman in prostitution. This suggests that men who have sex with a woman in prostitution do so for the first time when young or they will not do so at all. This finding is similar to that of research with men in the United Kingdom, where research has shown that if a man had not paid for sex by the age of 25 he was less likely to do so in the future [33,34]. The majority of men in the study who reported having had a transactional relationship or sex with a woman in prostitution were single or married and the prevalence of these practices in these groups of men did not differ. Men who were cohabiting were significantly more likely than married men to have done both of these practices. In South Africa’s conservative gender context, it is possible to interpret cohabiting as reflecting an unwillingness to commit to a partner, with such men being more likely to seek sex in a range of contexts where there is no expectation of commitment, especially where it is overtly or implicitly commodified. However we recognise as well that cohabiting may be a practice of marginalised very low income men who feel excluded from mainstream gender goals of establishing stable marriage unit. We recognise that this social meaning of cohabiting is different from that found in many higher income countries.

The prevalence of having had transactional sex (or sex in a provider role) disclosed by men is much higher than that reported by women, for example by Dunkle et al [6] (which was 21% with non-primary partners). There were some differences in the way the exposure was ascertained, but it is also possible that men are more likely to assert that involvement was due to an expectation of material gain. This may be in part a statement of provider prowess, obviously partly reflects expectations of women that sex partners will give them gifts, but it is also a commonly voiced complaint by men about women that they only are interested in men for what they can get from them. In this respect a greater likelihood of disclosing transactional sex may be associated with greater misogyny.

A strength of the study was the use of APDAs for data collection as these provided a confidential environment in which disclosure of anti-social and illegal behaviour could be enabled. Through removing the face to face component to interviews, APDAs greatly reduced the performative aspect of interview responses and so gave us more confidence that there would not have been a problem of over-reporting. Under or over-reporting, either deliberately, or through misconstruing women’s intentionality, is a recognised problem in research on transactional sex and stigmatised acts such as having sex with a woman in prostitution. It is impossible to estimate the magnitude of this in the study. Recall bias is a potential problem with any study of this nature. Self-completion of the questionnaire and the option of skipping questions resulted in some missing data on some items. We have not replaced missing values. Inevitably we had to keep this analysis within the constraints of the questions included in the questionnaire. It is unfortunate that these precluded us from obtaining other information about the men’s relationships or sexual acts, apart from this reported here. We did not retain information on the number of eligible men per household and so were not able to weight the analysis for this, but we have no reason to believe this would have made much difference to the estimates of association [35].

The findings have methodological importance as men from all racial groups clearly distinguish between adoption of a provider role/transactional sex and sex with a woman in prostitution and so it is methodologically important to maintain these distinctions in research. The most common provider role/ transactional sexual relationships were reported with main partners. This indicates that questions that focus on exchange in casual or once off partner relationships will miss the largest category of commodified sex or relationships.

Transactional sex or the male provider role, in particular, supports subtle understandings of gender inequality even while it seems more acceptable than the inequalities that are manifest in the commoditized arena of prostitution. By this reading, transactional sex assumes a resourced male in a supplicant sexual relationship with a woman who is assumed to be passive, but potentially can give or withhold sex. Transactional sex thus supports patriarchal sex roles (including a passive woman). On the other hand, transactional sex can also be considered as the arena in which women are more proactive, leaving the arena of marriage and familial negotiation, to pursue a more active role in exploring their material and sexual needs [23]. Similarly, for men with resources, it is the place where these resources are converted into heterosexualised masculinity in a way that avoids the public slur of *isoka lamanyala* (a man who takes womanising too far [11]: 165‐6]). It is precisely the possibility of these multiple readings of transactional sex that constitutes a resistance to any equation of the practice as a form of prostitution.

## Conclusions

Our findings suggest that research with women on the commodification of sex in Southern Africa may not translate easily when studying the role of men. Rather than viewing themselves comfortably as ‘transacting sex’, many men perceive an expectation that they should fulfil a provider role, and when they do so, they perceive themselves to be entitled to be rewarded with sex and obedience from women. This was seen to span all social categories, although predominantly to be an idea prevalent among Black African and Coloured men. This has important implications for intervention, as any efforts to work with men to reduce transactional sex should not focus on trying to stop a practice, but rather on reconfiguring how they see themselves as men. Whilst in some respects the acts and expectations within some encounters where men provide appeared to overlap with sex with a woman in prostitution, most men appeared to have made a distinction. Our findings suggest that whilst there appear to be similarities between having sex with a woman in prostitution and transactional relationships or sex (especially when a once off event), when viewed relationally, these are actually very distinct practices. Conflating them is sociologically inappropriate.

## Competing interests

The authors declare that they have no competing interests.

## Authors’ contributions

RJ was the principal investigator, led the study design, analysed the data and led the writing of the paper; RM contributed substantially to the questionnaire development and to framing and writing of the paper; YS contributed to the questionnaire development, was the project manager for the study and contributed to the interpretation of findings; KD contributed substantially to the study methodology and data collection, assisted in interpreting findings and drafting the paper; LPK assisted in interpreting findings and drafting of the paper. All authors contributed to the study design, interpretation of findings and drafting of the article. All authors read and approved the final manuscript.

## Pre-publication history

The pre-publication history for this paper can be accessed here:

http://www.biomedcentral.com/1471-2458/12/325/prepub
